# Emerging role of precision medicine in biliary tract cancers

**DOI:** 10.1038/s41698-018-0064-z

**Published:** 2018-10-03

**Authors:** James M. Bogenberger, Thomas T. DeLeon, Mansi Arora, Daniel H. Ahn, Mitesh J. Borad

**Affiliations:** 1 Division of Hematology/Oncology, Department of Medicine, Mayo Clinic, Scottsdale, AZ USA; 2 Department of Molecular Medicine, Mayo Clinic, Rochester, MN USA; 3 Mayo Clinic Cancer Center, Mayo Clinic, Phoenix, AZ USA

## Abstract

Biliary tracts cancers (BTCs) are a diverse group of aggressive malignancies with an overall poor prognosis. Genomic characterization has uncovered many putative clinically actionable aberrations that can also facilitate the prognostication of patients. As such, comprehensive genomic profiling is playing a growing role in the clinical management of BTCs. Currently however, there is only one precision medicine approved by the US Food and Drug Administration (FDA) for the treatment of BTCs. Herein, we highlight the prevalence and prognostic, diagnostic, and predictive significance of recurrent mutations and other genomic aberrations with current clinical implications or emerging relevance to clinical practice. Some ongoing clinical trials, as well as future areas of exploration for precision oncology in BTCs are highlighted.

## Introduction

**Fig. 1:**
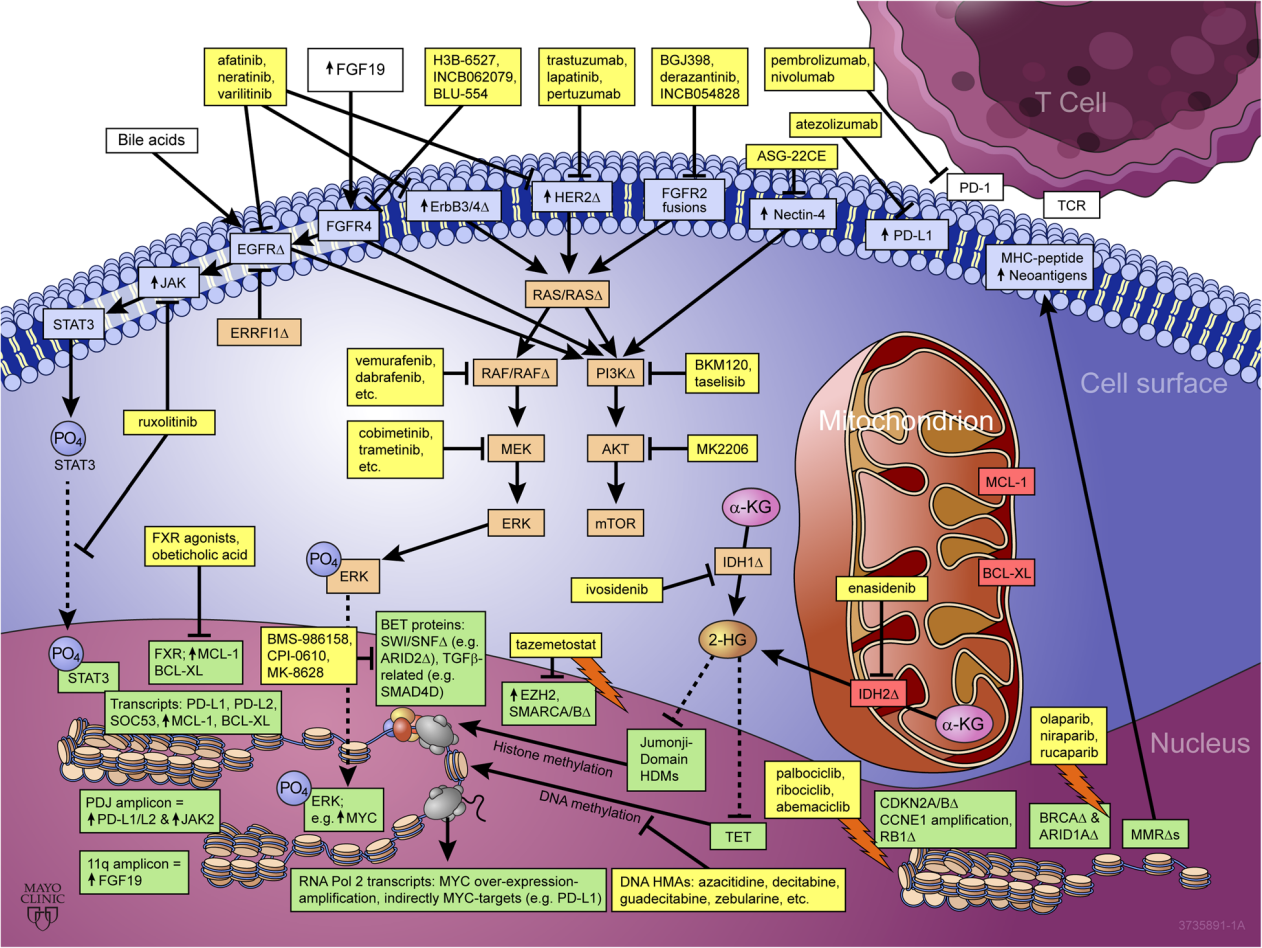
Emerging role of precision medicine in biliary tract cancers. Yellow boxes highlight US FDA-approved drugs and drugs undergoing clinical investigation as reviewed, with arrows indicating pathway/target activation and blocked lines indicating pathway/target inhibition. BTC targets/pathways discussed are shown in color-coded boxes according to subcellular localization, blue = cell surface, orange = cytsolic, red = mitochondrial, and green = nuclear. “↑” denotes over-expression, “Δ” denotes copy number abbreation and/or point mutation, a lighting bolt symbol denotes a synthetic lethal interaction between drug(s) and target(s) listed

Biliary tract cancers (BTCs) are a heterogeneous group of epithelial cell malignancies arising from distinct anatomical locations of the biliary tree (intrahepatic, perihilar, distal bile ducts or the gallbladder). BTCs are generally subtyped as intrahepatic cholangiocarcinoma (iCCA), extrahepatic cholangiocarcinoma (eCCA), and gallbladder carcinoma (GBC). Although surgery remains the only curative treatment option for BTCs, 5-year survival rates remain low, largely due to the advanced nature of these cancers at diagnosis and their aggressive underlying pathogenesis.^1^ Gemcitabine combined with cisplatin has emerged as a standard-of-care regimen for patients with inoperable disease.^2^ In recent years, a growing number of genomic studies have begun to uncover the molecular underpinnings of BTCs and suggest many potential treatments (Fig. 1). However, it should be noted that due to the relatively low incidence of BTCs, many essential reports thus far have investigated small-to-moderate cohorts associated with inherent sampling size and other biases. Thus, caution is necessary in interpreting these studies and formulating broader conclusions. Nonetheless, genomic studies of BTCs are ushering in a new era of precision therapy, already playing an emerging role in the treatment and prognostication of BTCs.

## Biomarkers of response to PD-1/PD-L1 immune checkpoint inhibitors

Microsatellite-instability (MSI) is a hypermutation phenotype that occurs in cells with defective mismatch repair (MMR), resulting in a significant increase in non-silent mutations per genome and concomitant increase in neoantigen presentation. The MSI/MMR phenotype arises via germline (Lynch Syndrome^3^) or somatic mutations of MMR genes (MLH1, MLH3, MSH2, MSH3, MSH6, PMS1, PMS2, POLD1, or POLE),^4^ silencing of MLH1 gene expression through promoter hypermethylation,^5^ epigenetic inactivation of MSH2,^6^ mutations in the promoter of PMS2,^7^ or downregulation of MMR genes by microRNAs.^8^ As the most high-profile testament of the potential of biomarker-guided clinical trials in oncology to date, a series of five uncontrolled, single-arm, multi-center clinical trials investigating pembrolizumab, an anti-PD-L1 immune checkpoint inhibitor monoclonal antibody, in MMR-deficient (dMMR)/MSI-high (MSI-H) advanced solid tumors (*N* = 149) has recently led to the first “tissue/tumor-agnostic” treatment approved by the U.S. FDA.^9^ Eleven of the 149 patients enrolled in these studies had BTCs. This small subset of BTCs showed a 27% overall response rate (ORR) as defined by RECIST v1.1, with a duration of response ranging from 11.6 to 19.6+ months.^9^ The ORR for this entire cohort of dMMR/MSI-H cases across all solid tumor types (90 colorectal and 59 non-colorectal) was 40%. dMMR/MSI occurs across all BTC subtypes, most frequently in iCCA with a prevalence of 10, and 5% prevalence for eCCA and GBC.^10^ Although there is currently no FDA-approved diagnostic test for this indication (indicated independently of PD-L1 expression), approval is based upon use of an MSI or MMR biomarker. MSI and MMR tests have been used in clinical practice for decades as a prognostic test for colorectal cancer.^11^ Polymerase chain reaction (PCR)-based measurement of the “Bethesda markers,” which include two monocucleotide and three dinucleotide repeats, is the “gold-standard” for MSI detection,^12^ and while immunohistochemistry for MMR proteins (MLH1, MSH2, PMS2, and MSH6) shows high concordance with PCR-based testing, cases of MSI with only missense mutations cannot be detected by immunohistochemistry. Tumor microdissection by a pathologist is critical for ensuring assessment of high tumor cellularity, as MSI diagnostics are insensitive below a purity of 20% tumor cells.

Beyond dMMR/MSI-H, tumor mutational burden (TMB) is emerging as a predictive biomarker for PD-1/PD-L1 immune checkpoint inhibitors. Higher TMB increases neoantigen presentation which promotes lymphocyte infiltration at the tumor periphery and microenvironment.^13^ Thus high TMB is associated with a higher likelihood of response to PD-1/PD-L1 immune checkpoint inhibitor therapy. A retrospective analysis of 151 solid tumor patients (primarily melanoma and non-small cell lung cancer) treated with PD-1/PD-L1 inhibitor therapy reported that 42% (16/38) of solid tumor patients with high TMB [defined as >20 mutations/megabase (Mb) determined by sequencing 1.2 Mb (~0.04% of the genome) and extrapolating to the whole genome] had an objective response, while 31% (21/67) of solid tumor patients with intermediate TMB (defined as 6–19Δ/Mb) had an objective response, and 2/46 patients with low TMB (defined as 1–5Δ/Mb) had an objective response.^14^ Prevalence of hypermutation varies by cancer type, and a study of 239 paired tumor/normal BTCs (137 iCCA, 74 eCCA and 28 GBCs) showed that 6% (14/239) were defined as high TMB using a threshold of >11.13Δ/Mb, which corresponded to a median 641 non-silent mutations in this subset of hypermutated BTCs.^15^ 10 of the 14 high TMB cases identified in this study were iCCAs (7%; 10/137), while two cases were eCCA (3%; 2/74) and two were GBCs (7%; 2/28). The remaining 94% of cases were defined by a significantly lower TMB <7.03 Δ/Mb and corresponded to a median number of non-silent mutations of 39, 35 and 64 for iCCA, eCCA and GBC, respectively.

The prevalence of PD-L1-positivity across BTCs is much higher than that of dMMR/MSI-H tumors, with clinically-actionable positively estimated to be at least 30–46%.^16,17^ Lower estimates of prevalence have likely emerged due to application of more stringent thresholds to define positivity, and the multiple assays used to define PD-L1 positivity, while none have been adopted as standard clinical practice. Multiple clinical trials investigating PD-1/PD-L1 immune checkpoint inhibitors specifically in BTCs are now ongoing including monotherapy trials (nivolumab, NCT02829918), and chemotherapy combinations evaluating gemcitabine and cisplatin (pembrolizumab, NCT03260712; nivolumab, NCT03101566) (Table 1). In the fully-enrolled but ongoing KEYNOTE-028 study of monotherapy pembrolizumab (NCT02054806), 42% (37/89) of the BTC patients screened were defined as PD-L1-positive tumors utilizing a threshold of ≥1% of tumors cells or infiltrating lymphocytes expressing PD-L1. The preliminary ORR for the 24 BTC patients enrolled was reported at 17% (all durable PRs with treatment ongoing 40–42+ weeks at the time of reporting), with a disease control rate (DCR) of 33% (8/24).^18^

**Table 1:** Highlighted ongoing clinical trials evaluating biliary tract cancers

Target(s)	Investigated drug(s)/arm(s)	Enrollment criteria	National Clinical Trial (NCT) identifier	Putative precision oncology application for BTCs
PD-1	nivolumab monotherapy	PD-L1-positive BTCs	NCT02829918	PD-L1-positive BTCs, LELCC
PD-1 + cytotoxic chemo	(nivolumab or pembrolizumab) with gemcitabine + cisplatin	PD-L1-positive BTCs	NCT03101566, NCT03260712,	PD-L1-positive BTCs, LELCC
pan-FGFR1/2/3	INCB054828 monotherapy	FGFR2 translocations	NCT02924376	FGFR2 translocations
pan-FRFR1/2/3	ARQ087/derazantinib monotherapy	iCCA or combined HCC/CCA with FGFR2 translocations	NCT03230318	FGFR2 translocations
FGFR4	H3B-6527 or INCB062079 monotherapy	Unselected CCA	NCT02834780, NCT03144661	FGF19 amplification
IDH1	ivosidenib monotherapy versus placebo	IDH1-mutant CCA	NCT02989857	IDH1-mutant CCA
HER2	trastuzumab + pertuzumab	BTCs with HER2 amplifications, over-expression, or activating mutations	NCT02091141 (one arm of “My Pathway” Study)	BTCs with HER2 amplifications, over-expression, or activating mutations
pan-HER + /- SERD or cytotoxic chemo	niratinib monotherapy, niratinib + fulvestrant or niratinib + paclitaxol	HER2, ERBB3, EGFR-mutated or EGFR-amplified BTCs	NCT01953926	HER2, ERBB3, EGFR-mutated or EGFR-amplified BTCs
pan-HER + cytotoxic chemo	afatinib + capecitabine	Unselected BTCs	NCT02451553	ERBB3- and ERBB4-alterations
pan-HER	ASLAN001/varlitinib montherapy	Unselected BTCs	NCT02609958	ERBB3- and ERBB4-alterations
pan-HER + cytotoxic chemo	ASLAN001/varlitinib with gemcitabine + cisplatin	Unselected BTCs	NCT02992340	ERBB3- and ERBB4-alterations
pan-HER + cytotoxic chemo	ASLAN001/varlitinib + capecitabine versus capecitbine	Unselected BTCs	NCT03093870	ERBB3- and ERBB4-alterations
Bromodomain/BET Proteins	BMS-986158	Multiple advanced solid tumors, unselected	NCT02419417	MYC activation and/or amplification
Nectin-4	ASG-22CE	Nectin-4 expressing solid tumors	NCT02091999	GBCs evaluated for Nectin-4 expression, and PI3K/AKT/mTOR-mutated BTCs

*AKT* proto-onogene C-Akt, *BET* bromo- and extra-terminal domain faimly, *BTCs* biliary tract cancers, *CCA* cholangiocarcinoma, *EGFR* epidermal growth factor receptor, *ERBB3* human epidermal growth factor receptor 3, *ERBB4* human epidermal growth factor receptor 3, *FGF19* fibroblast growth factor 19, *FGFR1/2/3/4* fibroblast growth factor receptor 1/2/3/4, *GBCs* gallbladder cancers, *HCC* hepatocellular carcinoma, *HER2* human epidermal growth factor receptor 2, *iCCA* intrahepatic cholangiocarcinoma, *IDH1* isocitrate dehydrogenase 1, *LELCC* lymphoepithelioma-like cholangiocarcinoma, *mTOR* mammalian target of rapamycin, *MYC* proto-oncogene C-Myc, *PD-1/PD-L1* programmed cell death protein/ligand 1, *PI3K* phosphatidylinositol 3-kinase, *SERD* selective estrogen receptor downregulator

A subset of lymphomas, triple-negative breast cancers, and Epstein Barr Virus-positive gastric cancers are associated with genomic amplifications of a region of chromosome 9 containing PD-L1, PD-L2, and JAK2 loci termed the PDJ amplicon.^19–21^ These malignancies harboring the PDJ amplicon exhibit increased levels of PD-1 ligand and JAK2, while JAK2 inhibition was shown to decrease PD-1 ligand expression. A rare BTC subtype known as intrahepatic lymphoepithelial-like cholangioncarcinoma (LELCC), frequently associated with Epstein Barr Virus infection, and a higher prevalence in Asia, is reported to express high levels of PD-L1.^22^ It is compelling to speculate that LELCC cases may also harbor the PDJ amplicon. PD-L1-positive BTCs are clear candidates for PD-1/PD-L1 checkpoint inhibitor therapy, although a role for JAK2 inhibition in combination with PD-1/PD-L1 checkpoint inhibitor therapy is also intriguing to consider for this subset of BTCs. A phase 1 study exploring this concept with combined pembrolizumab and JAK inhibitor ruxolitinib in triple-negative breast cancer has been initiated (NCT03012230).

## FGF signaling aberrations

FGFR2 aberrations are almost exclusively observed in iCCA, and occur at a frequency of approximately 15% with the vast majority of FGFR2 aberrations being fusions and thus also amenable to clinical detection by fluorescent in situ hybridization (FISH) break-apart assays.^23^ FGFR2 rearrangements are associated with increased overall survival (OS) in the post-resection setting (median 123 months vs. 37 months for cases without FGFR2 rearrangements).^24^ Patients with FGFR aberrations may have superior OS with FGFR-targeted therapy as compared to standard regimens.^25^ FGFR2 fusions activate MAPK signaling and typically do not co-occur with KRAS/BRAF mutations.^23,26^ BGJ398, a pan-FGFR small molecule inhibitor with 0.9, 1.4, and 1.0 nM potency for FGFR1/2/3 respectively in cell-free assays and >40-fold selectivity over FGFR4,^27^ has been studied in a phase 2 trial in advanced CCA patients resistant to frontline gemcitabine-based therapies.^28^ In 61 patients (*N* = 48 FGFR2 fusions, *N* = 8 FGFR2 mutations, *N* = 3 FGFR2 amplifications; with >1 type of FGFR2 aberration detected in 3 patients) the ORR, all partial responses (PRs), was 15%, with 75% of patients experiencing some disease control and a median progression-free survival (PFS) of 5.8 months. Four patients carried FGFR3 amplifications but none responded to BGJ398. Dose modifications were required for many patients, although AEs were mostly reversible. The most common AE was hyperphosphatemia (72%), with 25% of patients experiencing grade 3 or 4 hyperphosphatemia.^29^ Hyperphosphatemia may be an on-target effect related to FGF23-regualted phosphate homeostasis.^30,31^ INCB054828, a pan-FGFR1/2/3 inhibitor with half-maximal activity of 3–50 nM in FGFR-dependent cell viability assays with >30-fold selectivity over FGFR-independent cell lines, has reported preliminary clinical activity in a small cohort of CCA patients,^32^ and has now progressed into phase 2 development (NCT02924376). ARQ087 (derazantinib) is a pan-FGFR inhibitor which inhibits FGFR2 most potently, with 4.5, 1.8, 4.5, and 34 nM potency for FGFR1/2/3/4, respectively in cell-free assays.^33^ A phase 1/2 study of ARQ087 (derazantinib) in advanced solid tumors reported only 5% grade 1 hyperphosphatemia.^34^ An expanded FGFR2-abberrant CCA cohort (*N* = 35) from this study reported a PR rate of 20% (6/35) and 49% stable disease (17/35). Abnormal LFTs and asthenia were observed in 20% of this cohort with 6% being grade 3/4.^35^ Based on this preliminary clinical activity, a single-arm phase 3 study of derazantinib in refractory iCCA with FGFR2 fusions is ongoing (NCT03230318).

Development of FGFR2 therapies is currently challenged by primary resistance and often limited response durability. Acquired resistance to FGFR2-targeted therapy via concordant evolution has been reported to manifest as mutations in the kinase domain of FGFR2 affecting drug binding.^36^ Using a liquid biopsy approach to assess circulating cell-free DNA,^37^ three FGFR2 fusion iCCA patients responding to BGJ398 acquired a V564F mutation at the time of progression, although two patients also acquired several additional polyclonal FGFR2 mutations exclusive to progression. Subsequent use of alternative FGFR2 inhibitors may lead to responses after progression, analogous to EGFR or ALK inhibitors in lung cancer.^36^ Additionally, divergent evolution mechanisms, such as PTEN aberrations, may also play a role in acquired resistance to FGFR2-targeted therapies.^36^ Thus, therapeutic combination strategies targeting FGFR2 and PTEN/PI3K/AKT may be speculatively intriguing to increase response duration, and/or overcome primary resistance in some FGFR2 fusion cases with co-occurring PTEN/PI3K alterations.

Amplicons of chromosome 11q containing of FGF19, an exclusive ligand of FGFR4 activating MAPK and JNK signaling in hepatocytes,^38,39^ have been reported in CCA.^40^ FGF19 amplification has been proposed as a predictive biomarker for sorafenib response in hepatocelluar carcinoma (HCC),^41^ despite a lack of relevant FGFR inhibition by sorafenib, suggesting relevance of downstream sorafenib inhibition of RAF1/BRAF in this context. Sorafenib monotherapy has shown limited activity in advanced iCCA with PFS of 2.3–3.2 months,^42,43^ although fourth line sorafenib treatment of a single CCA case was reported to yield OS >4 years,^44^ suggesting the possibility that a rare underlying genomic context of CCA might benefit from sorafenib. Several FGFR4 inhibitors are now being evaluated clinically. H3B-6527 and INCB062079 are being investigated in phase 1 trials including CCAs (NCT02834780 and NCT03144661, respectively), while BLU-554 is being investigated in a phase 1 evaluating HCC only (NCT02508467).

## Isocitrate dehydrogenase (IDH) mutations

IDH point mutations result in high-level accumulation of the oncometabolite 2-hydroxygluterate which inhibits enzymes that utilize alpha-ketogluterate as a cofactor, such as DNA methyltransferase TET2 and Jumonji C domain containing histone demethylases, leading to DNA and histone hypermethylation, respectively, and resultant dysregulation of gene expression.^45,46^ IDH mutations occur mostly in iCCA at ~20% frequency.^47,48^ IDH1 mutations occur primarily at the R132 codon and are most frequent, although IDH2 mutations at the R172 codon occur at ~half the frequency of IDH1R132Δ point mutations. Conflicting results have been reported for the prognostic significance of IDH mutations.^48–50^ The initial target population for clinical trials of IDH inhibitors is unresectable and/or metastatic disease, and prognostic significance may vary based on the precise clinical setting. In addition to disease presentation considerations, we speculate that the pathogenesis and prognostic impact of IDH mutations is likely modulated by co-occurring driver mutations. AG-221 (enasidenib) is now approved for treatment of acute myeloid leukemia (AML) with IDH2 mutations, although enasidenib has not yet been tested in CCA. AG-120 (ivosidenib) showed somewhat promising clinical activity in preliminary studies with an ORR 6% and DCR of 62%. A randomized, placebo-controlled phase 3 trial with PFS as the primary endpoint is currently investigating ivosidenib in IDH1-mutant BTCs (NCT02989857). Hypomethylating agents (HMAs) such as azacitidine are de facto standard-of-care therapies for elderly AML unfit for induction therapy, and a clinical trial evaluating azacitidine in combination with enasidenib or ivosidenib in IDH-mutant AML is ongoing (NCT02677922). An HMA and IDH inhibitor combination may have utility in CCA based on analogous scientific rationale that HMAs will universally synergize with IDH inhibitors in reversing pre-established aberrant DNA methylation.

## ErbB/HER pathway alterations

Multiple aberrations within the ErbB/HER signaling pathway have been described in BTCs, including ERBB1/EGFR mutations and amplifications, ERRFI1 mutations, ERBB2/HER2, ERBB3, and ERBB4 amplifications and mutations, while HER signaling is the most frequently mutated pathway in GBC. EGFR amplifications occur in 19–31% of iCCA/eCCA and are associated with poor prognosis,^51,52^ while somatic EGFR mutations were reported in 4% (3/51) of GBC.^53^ A phase 3 study of erlotinib added to gemcitabine and oxaliplatin in advanced BTCs unselected for ErbB/HER pathway aberrations failed to show an improvement in PFS over the control arm.^54^ A mutation in ERRFI1 (E384X), a direct inhibitor of EGFR, was identified in an iCCA case which responded rapidly to erlotinib.^55^ ERRFI1 has not been broadly evaluated, thus incidence and prognostic significance are unknown. HER2 gene amplifications and mutations are most prevalent in GBC and eCCA (10–15%), and are less common in iCCA.^25,56^ Prognosis for HER2 aberrations are unknown for BTCs, but are well known to have aggressive phenotypes in breast and gastric cancers. Currently, only one case series has been published for HER2-targeted therapy (trastuzumab, lapatinib, or pertuzumab) in BTCs (*N* = 9 GBCs; *N* = 5 iCCAs).^53^ Amongst the GBCs with HER2 aberrations evaluated, responses included one complete response, four PRs, and three stable disease, with a median duration of response of 40 weeks (8+ to 168 weeks, with three patients continuing to respond at the time of this report), while there were no radiological responses amongst the 5 iCCAs with HER2 aberrations evaluated. The multi-arm, biomarker-driven “My Pathway” study (NCT02091141) includes a trastuzumab plus pertuzumab arm evaluating BTCs with HER2 amplifications, over-expression, or activating mutations. ERBB3 mutations occurred in 12% (6/51) of BTCs, while ERBB4 mutations occurred in 4% (2/51).^53^ Pan-HER inhibitors such as afatinib, neratinib and varlitinib may play a role in ERBB3 and ERBB4 altered BTCs where more selective EGFR or HER2 inhibitors are not applicable. Afatinib is being assessed in an unselected phase 1 trial (NCT02451553). The investigational pan-HER inhibitor ASLAN001 (varlitinib) is being evaluated in a single-arm monotherapy study (NCT02609958), in a gemcitabine and cisplatin single-arm combination study (NCT02992340), and in a randomized phase 3 comparing varlitnib plus capecitabine versus capecitabine alone (NCT03093870), all actively enrolling BTCs specifically without HER biomarker selection. Neratinib is being investigated as monotherapy and combined with fulvestrant or paclitaxel in an open-label efficacy study of HER2, ERBB3, or EGFR-mutated/-amplified solid tumors including BTCs (NCT01953926).

## MAPK signaling

ErbB family receptors often operate through MAPK signaling, while mutations in MAPK signaling also occur in BTCs. KRAS mutations are the most common mutations in iCCA with a prevalence of ~19% but also occur across all BTC subtypes,^57^ and are associated with a poor prognosis.^58^ Targeting KRAS directly has proven difficult, thus strategies for targeting KRAS-mutant BTCs require further efforts. BRAF mutations occur in 3–5% of iCCA cases, although no clinical trial has assessed a BRAF inhibitor in CCA. MEK inhibitors have shown some limited single-agent clinical activity in BTCs.^59^ Several clinical trials are evaluating PD-1/PD-L1 antibodies in combination with BRAF/MEK inhibitors in solid tumors such as colorectal and melanoma (NCT02788279, NCT02902029, NCT02858921, NCT01988896) based on data demonstrating MEK inhibition increases antigen presentation,^60^ with preliminary safety and activity reported.^61^ Based on the aforementioned single-agent PD-L1 responses in BTCs, relevance of MAPK signaling, activity of single-agent BRAF and MEK inhibitors, and demonstrated safety of the triplet combination in solid tumors, such combination(s) are logical to investigate in PD-L1-positive BTCs. Preclinical data suggests that PI3K-axis signaling inhibition with an AKT inhibitor can overcome acquired resistance to MEK inhibition in CCA.^62^ Thus, MEK and AKT inhibitor combinations in BRAF-mutant iCCA, and MEK, AKT, and PD-1/PD-L1 inhibitor triplet combinations in PD-L1-positive BTCs can be envisioned.

## MET

High c-Met expression was observed in 12% of iCCA and 16% of eCCA and portends a poor prognosis, while MET amplifications are observed in 2–7% BTCs.^63^ Cabozantinib, a multi-kinase small molecule inhibitor that potently inhibits MET^64^ and is FDA-approved for advanced renal cell carcinoma and thyroid cancer, showed limited activity and significant toxicity in a study of unselected CCA, and response did not correlate with MET expression.^65^ However, MET amplification may be a more potent driver than MET expression.

## PI3K/AKT/mTOR

Mutations in the PI3K/AKT/mTOR signaling pathway frequently occur in eCCA (40%) and iCCA (25%), including FBXW7, PI3KCA, PTEN, NF1, NF2, PIK3R1, STK11, TSC1, and TSC2, and are associated with a worse prognosis.^58,66^ Mutations in PIK3CA are frequent in GBC (8–13%).^57,67^ PI3K/AKT/mTOR often signals downstream of ErbB/HER and preclinical studies suggest activity of PI3K/AKT/mTOR inhibition in BTCs,^68,69^ although clinical studies are scarce. PI3K inhibitors in clinical development such as BKM-120 and taselisib may be relevant to BTCs. A clinical study of AKT inhibitor MK2206 was terminated due to futility.^70^ While PI3K/AKT/mTOR inhibitor monotherapy may have clinical utility for a subset of BTCs with pathway alterations, further development of PI3K/AKT/mTOR inhibitors will require biomarker selection of patients, and combination strategies that consider co-occurring drivers (e.g., IDH mutations frequently occur with PIK3CA mutations), as well as acquired resistance (e.g., combination with MEK inhibitors in BTCs cases with aberrant MAPK signaling were acquired resistance may emerge via PTEN/PI3K^62^). Nectin-4, a cell adhesion molecule with diverse functions, was over-expressed in 63% (43/68) of GBC cases, while activation of PI3K/AKT was involved in the oncogenic function of Nectin-4 and PI3K inhibitors impaired Nectin-4-mediated GBC proliferation and motility.^71^ Thus, evaluation of Nectin-4 expression in PI3K/AKT/mTOR-mutated cases could be considered. A phase 1 study of a Nectin-4 antibody drug conjugate ASG-22CE^72^ is enrolling solid tumor patients with confirmed Nectin-4 expression (NCT02091999).

## Rb-cell cycle dysregulation

CDKN2A/B genes are frequently mutated or amplified in iCCA (7–27%),^25,73^ eCCAs (17–47%)^25,26^ and GBC (19–26%),^25,74^ while silencing via promoter methylation is also observed in BTCs.^74,75^ In unresectable BTCs, the joint status of CDKN2A and p53 has been associated with a poor prognosis.^76^ CCND1/3 mutations are observed across all BTCs (4–14%), as well CCNE1 mutations/amplifications (4–5% in eCCA/GBC) and RB1 mutations (1–7%).^15^ CDKN2A is a well-known inhibitor of CDK4/6 and MDM2. Three CDK4/6 inhibitors are now FDA-approved for use in breast cancer treatment (palbociclib, ribociclib, and abemaciclib) that could be considered for BTCs.

## Genome stability/DNA repair

Mutations of p53 are common across all BTCs (18–43%) and portend a poor prognosis.^58,73,77^ p53 mutations should be evaluated with precisely defined recurrent co-mutations using unbiased functional studies to identify potential therapeutic strategies. MDM2 mutations occur across all BTCs at 5–7%.^15^ BRCA1/2 mutations occur at 1–7% across BTCs (most frequently BRCA2 in GBC) and are candidates for PARP inhibition, which is synthetic lethal with BRCA mutations (olaparib, niraparib and rucaparib are FDA-approved for ovarian and breast cancer indications). A study reporting on 18 BRCA1/2-mutated CCAs found that 28% (5/18) carried germline mutations.^78^ Sustained responses to PARP inhibitors have been reported for BTCs.^78,79^ Additionally, commonly-occurring ARID1A mutations (see subsequent section on chromatin modifiers) are also reported to be synthetic lethal with PARP inhibition.^80^

## Chromatin modifiers

In addition to the aforementioned IDH mutations, additional chromatin-modifying enzymes are recurrently mutated or altered by copy number aberrations in BTCs (alterations in epigenetic regulators occur in about one-third of BTCs), including ARID1A (11–36% in all BTCs; most frequently iCCA), ARID1B (5%; eCCA), ARID2 (4–18%; all BTCs, most frequently GBC), KDM4A (7%; GBC), KDM5D/-6A/-6B and UTY (1–4%; iCCA/eCCA), TET1/2/3 (5–11%; iCCA/GBC), PBRM1 (7–21%; iCCA/GBC), SMARCAD1/A2/A4 (1–3% each; iCCA/eCCA) and KMT2D/MLL2 and KMT2C/MLL3 (2–11%; all BTCs, most frequently GBCs-), ASXL1 (2–3%; iCCA/eCCA), as well as the deubiquitinating enzyme BAP1 (8–32%; iCCA, less frequently in eCCA), which while not a direct chromatin modifier is associated with a DNA hypermethylation phenotype in iCCA and other tumor types.^15,26,58,73,81,82^ Promoter DNA methylation of at least one tumor suppressor gene occurs in 85% of CCA cases.^83^ Additionally, novel non-coding promoter mutations have been reported to be associated with histone methylation (H3K27me3) modulation of CCA.^84^ Using a novel method named FIREFLY, for finding regulatory mutations in gene sets with functional dysregulation, to identify and define gene sets with altered transcription factor binding due to promoter mutations, researchers identified four gene sets in CCA, two of which contained subsets of target genes epigenetically silenced by the PRC2 complex via H3K27me3 in specific contexts. CCA was also defined by two distinct DNA hypermethylation phenotypes, with one subgroup showing concurrent down-regulation of DNA demethylation enzyme TET1 and upregulation of histone methyltransferase EZH2 suggesting an epigenetic role in establishing the DNA hypermethylation phenotype of this subgroup, while the other subgroup had IDH and/or BAP1 mutations.^84^ EZH2 inhibitors are now in clinical development (e.g., tazemetostat for some EZH2, SMARCB1, or SMARCA4-mutated cancers, NCT03213665) and could be considered for the EZH2/TET1 hypermethylated subgroup, or for SMARCA/B-mutated BTCs. DNA hypermethylation alone is not typically a sufficient driver or vulnerability of BTCs in and of itself, although it is compelling to speculate that HMAs could form the backbone of combination therapies for hypermethylated CCAs. For example, in a study of 260 Japanese BTC cases, BAP1 mutations most commonly occurred with FGFR2 fusions,^15^ thus FGFR2-targeted therapy could be investigated in combination with a HMA in patients with these co-occurring mutations. Recurrent mutations in SWI/SNF chromatin modifying complex components (ARID1A/B, ARID2, PBRM1, and SMARCA2/A4/AD1), as well as mutations in TGFβ-related genes [most frequently SMAD4 (4–10% across BTCs), but also less frequent mutations in TGFBR1/2, ACVR2A and FBXW7], are known to upregulate MYC, and MYC amplification in BTC appears to be mostly mutually exclusive with alterations in TGF-β and SWI/SNF signaling components, suggesting synonymous function(s).^15^ Thus, BET inhibitors, which block MYC transcription by preventing chromatin-dependent signal transduction to RNA polymerase 2,^85^ represent a compelling monotherapy strategy to target BTCs with activated/amplified MYC, and could speculatively form the backbone of combination strategies for BTCs with MYC activation and/or amplification. MYC regulates expression of PD-L1, while BET inhibitors were reported to down-regulate PD-L1 expression, which may have implications as immunotherapy or for immunotherapy-based combinations.^86^ There are several BET inhibitors currently being investigated in clinical trials, e.g., BMS-986158 in advanced solid tumors (NCT02419417), CPI-0610 in various myeloid malignancies (NCT02158858), and MK-8628 in hematological malignancies (NCT02698189).

## Apoptosis

Anti-apoptotic proteins BCL-XL and MCL-1 are both thought to have underlying biological relevance in BTCs, while BCL-2 was not expressed in normal or malignant biliary epithelium.^87^ MCL-1 is frequently amplified in iCCAs (16–21%),^58,73^ and is also overexpressed via epigenetic silencing of the SOCS3 promoter by DNA hypermethylation,^88^ and upregulated by bile acids downstream of EGFR.^89^ SOCS3 levels are inversely correlated with STAT3 phosphorylation levels via a feedback loop regulated by Janus kinases that control expression of both MCL-1 and BCL-XL. In another preclinical study, FXR agonists were shown to have the capacity to down-regulate BCL-XL via inhibition of STAT3 phosphorylation,^90^ and would therefore also have the capacity to down-regulate MCL-1. Thus HMAs, JAK inhibitors and FXR agonists all represent compelling therapeutic strategies in cases of MCL-1 over-expression, possibly including amplifications, as well as in apoptosis-targeting combination therapies. There are several HMAs and JAK inhibitors FDA-approved for other indications (and several additional candidates in clinical testing), while FXR agonist obeticholic acid is FDA-approved for the treatment of primary biliary cholangitis, and several FXR agonists are also undergoing clinical investigation for primary scelerosing cholangitis and nonalcoholic steatohepatitis.

## Consideration of co-mutations/comprehensive genomic-epigenomic landscapes

It will be absolutely critical to move beyond targeting individual mutations to advance precision oncology in BTCs, especially for more indolent drivers such as FGFR2 fusions and IDH mutations that co-operate with additional mutations, copy number aberrations, or epigenomic alterations. In BTCs, mutations in epigenetic regulators often occur with growth-promoting alterations, such as the aforementioned example of FGFR2 aberrations with BAP1 mutations, and KRAS, EGFR, or PIK3CA with IDH mutations.^15^ Thus therapeutic strategies that target both drivers, such as combination of an HMA with an FGFR2 inhibitor, or RAF, EGFR, AKT inhibitors with IDH inhibitors are compelling in these contexts, respectively. In one study, all 22 KRAS-mutant CCAs evaluated co-occurred with CDKN2A promoter hypermethylation, suggesting that a MAPK pathway inhibitor with a CDK4/6 inhibitor is a compelling combination strategy for KRAS-mutant CCA with epigenetically silenced CDKN2A.^91^ As highlighted herein, iCCA, eCCA, and GBC show unique prevalence for specific mutations, although the basis for this is unknown. Some researchers have proposed that differences in mutational prevalence are related to differences in embryonic origin, and it has also been proposed that micro-environmental exposure based on differences in tissue function may also contribute to prevalence differences. Thus, combinations should also be considered and designed in a tissue/BTC-subtype-specific manner.

## Liquid biopsy/circulating cell-free DNA detection in diagnosis, treatment monitoring, and surveillance

As exemplified by the aforementioned study detecting FGFR2 mutations,^36^ liquid biopsy represents a powerful approach for discovering mechanism(s) of drug resistance, and monitoring tumor evolution analogous to what is currently performed for EGFR mutations in lung cancer.^92^ Liquid biopsy is suitable for identifying clinically-actionable alterations, and there is a high concordance between tissue and plasma measurements.^93^ Liquid biopsy is easy to implement and non-invasive, and thus it will be used more and more in the precision oncology setting. Intrapatient tumor sample heterogeneity has necessitated multi-region sequencing previously, although liquid biopsy yields a uniform, quantitative plasma signal which represents an advantage in detecting intrapatient tumor heterogeneity as well. Physicians and patients will not, and often cannot, extensively/serially biopsy, thus liquid biopsy will be a critical precision oncology tool by facilitating serial sample collection for biomarker discovery, treatment monitoring, and disease surveillance in BTCs.

## Preclinical models

Beyond consideration of individual clinically actionable mutations, further progress will be largely dependent on developing treatment strategies to address recurrent co-mutations and ultimately global genome profiles. Preclinical models for investigating co-mutations, and potentially even more complex precisely-defined genomic context(s), such as comprehensive inclusion of mutations with clinically-observed gene expression changes (e.g., HER2Δ or MET overexpression) and/or broader epigenetic dysregulation (e.g., BAP1), will be critical in facilitating clinical-translational progress of the rapidly emerging genomic knowledge of BTCs. Serial sampling studies utilizing liquid biopsy will be powerful in constructing preclinical models of acquired drug resistance. Organoid models can facilitate the culture of primary samples with preservation of in vivo tumor characteristics, correlating well with clinical characteristics. Importantly organoid models can facilitate the efficient and cost-effective preclinical characterization of targeted therapies and combinations thereof.^94^ In addition to testing logically-deduced drug selection, unbiased functional studies, such as Genome-Scale CRISPR-Cas9 Knock-Out screening,^95^ will be powerful preclinical tools to advance precision medicine in more complex models where known genomic alterations may be currently “undruggable,” and where the best targeting strategies may not be obvious, e.g., p53-mutated or KRAS-mutated context(s). Where adequate resources are available, and where preclinical data justify, patient samples can also be assessed in patient-derived xenograft and/or transgenic animal models used.

## Clinical trial design for BTCs in the era of precision medicine

**Fig. 2:**
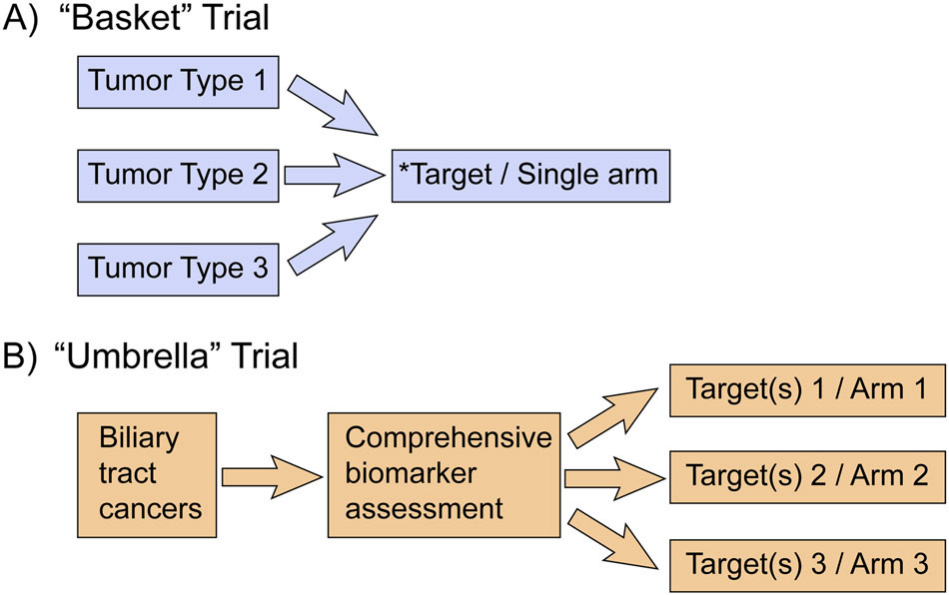
Clinical trial design for biliary tract cancers in the era of precision medicine. **a** “Basket” trial design vs. **b** “umbrella” trial design. *Preferable for BTC targets occurring at low prevalence (<5%)

In terms of clinical investigation, there is a tension in the BTC field between pursuing empirical approaches versus knowledge-based approaches, which represents a huge problem particularly in the adjuvant setting as compared to the advanced setting. The BILCAP study is the first study in the adjuvant setting to enroll a sufficient number of BTC patients to demonstrate that chemotherapy can significantly improve OS. Capecitabine improved OS by 15 months over surgery alone (53 vs. 36 months).^96^ Single-drug, single-trial, company-driven trials currently predominate the clinical trial landscape. This clinical trial design is sub-optimal for BTC patients, as well as the progress of precision oncology. A single, continuous “umbrella” trial, that can be used for registration studies, conducted using a single comprehensive assay that can identify all known biomarkers and facilitate assignment of patients to multiple corresponding treatment arms (arms which can be dynamically added or terminated as necessary) represents a much more appropriate patient-centric approach, as compared to antiquated sponsor-centric approaches (Fig. 2). A French trial called ONCOBIL is genotyping patients with malignant biliary stricture as compared to patients treated for benign biliary diseases (*N* = 50 each, NCT02893085), but does not feature assignment to corresponding treatment arms. For very low prevalence alterations such as NTRK or ROS1 fusions^97,98^ a more relevant design may be the “basket” or tumor-agnostic trial where approval is based on the target and not by the disease, as exemplified by PD-1 inhibitors for MSI/MMR, and likely forthcoming approvals for LOXO-101 (larotrectinib^99^) and RXDX-101 (entrectinib^100^) based on NTRK and NTRK/ROS1 fusions. However, not all mutations will be amendable to tumor-agnostic monotherapy development approaches, as many mutations will undoubtedly have distinct underlying biology and thus distinct outcomes may be observed in different tumors, as exemplified by MEK and BRAF inhibitor development.

## Conclusions

While treatment of MSI-H/dMMR tumors with pembrolizumab is currently the only targeted, biomarker-based therapy FDA-approved for BTCs, there are many potentially actionable aberrations in BTCs, and comprehensive genomic profiling is highly recommended in the management of BTCs. Additional biomarker-based treatments such as NTRK/ROS1-targeted therapies, albeit very low prevalence in BTCs, and FGFR-targeted therapies, are likely candidates for near-term regulatory approvals. There are several targeted therapies FDA-approved for other indications (e.g., HER2-targeted agents) with potential relevance for precision oncology application in BTCs that could possibly be considered for off-label use on a case-by-case basis. Importantly, there are also several biomarker-driven and unselected clinical trials for many of these FDA-approved agents to expand into BTCs, as well as for novel targeted therapies, that should be watched and considered for enrollment. Biomarker-driven umbrella or basket trials will be of high interest to BTC research efforts, as well as facilitating the development of novel targeted agents and combinations thereof. Many of the mutations/aberrations observed in BTCs are often indolent drivers alone (e.g., IDH or FGFR2), and even where such drivers may be significantly beneficial to target as monotherapy, combination therapy targeting two or more drivers is likely to yield deeper and more durable responses. Well-designed preclinical models, that recapitulate in vivo properties and thus can accurately interrogate precise genomic contexts to derive and test such combination therapies, will be paramount in moving beyond empirical therapy into a new era of precision therapy for BTCs.
